# Mycobiome dysbiosis and genetic predisposition to elevated IL-17A contribute to fibrosis in MASLD

**DOI:** 10.1016/j.jhepr.2025.101721

**Published:** 2025-12-23

**Authors:** Nadja Thielemann, Sara Leal Siliceo, Monika Rau, Annika Schöninger, Nathalie Reus, Alexander M. Aldejohann, Aia Shehata, Isabell S. Behr, Natalie E. Nieuwenhuizen, Michaela Herz, Heike M. Hermanns, Mohammad Mirhakkak, Jürgen Löffler, Thomas Dandekar, Kerstin Hünniger-Ast, Ronny Martin, Gianni Panagiotou, Andreas Geier, Oliver Kurzai

**Affiliations:** 1 Institute for Hygiene and Microbiology, University of Würzburg, Würzburg, Germany; 2 Microbiome Dynamics, Leibniz Institute for Natural Product Research and Infection Biology - Hans Knöll Institute, Jena, Germany; 3 Department of Medicine II, Division of Hepatology, University Hospital Würzburg, Germany; 4 Research Group Fungal Septomics, Leibniz Institute for Natural Product Research and Infection Biology - Hans Knöll Institute, Jena, Germany; 5 Department of Internal Medicine II, University Hospital Würzburg, Germany; 6 Functional Genomics & Systems Biology, Department of Bioinformatics, University Würzburg, Germany; 7 National Reference Center for Invasive Fungal Infections, Leibniz Institute for Natural Product Research and Infection Biology- Hans Knöll Institute, Jena, Germany; 8 Faculty of Biological Sciences, Friedrich Schiller University, Jena, Germany; 9 Department of Medicine, The University of Hong Kong, Hong Kong Special Administrative Region of China

**Keywords:** mycobiome, intestinal fungi, Th17 signaling, IL-17A, MASLD, MASH, *Candida*, liver fibrosis, liver inflammation

## Abstract

**Background & Aims:**

Metabolic dysfunction-associated steatotic liver disease (MASLD) is the leading cause of chronic liver disease in Western countries. Progression to metabolic dysfunction-associated steatohepatitis (MASH) occurs when fat accumulation in the liver triggers inflammatory processes including T helper 17 cell (Th17) activation. We aimed to investigate the role of intestinal fungi in MASH-mediating Th17-associated signaling.

**Methods:**

Blood samples from patients with MASLD (n = 451), including 141 with histology-proven MASH, were genotyped for *IL17A* rs2275913. Microbiome composition was assessed by ITS1 and 16S rRNA sequencing of stool samples from patients with MASLD (n = 221), including 79 with histology-proven MASH, as well as 25 healthy controls. Highly abundant fungal species identified in patients with MASH were used to stimulate *IL17A* rs2275913–genotyped T cells *ex vivo*, and cytokine levels were measured (n = 9 per genotype). Th17/resting regulatory T cell (Th17/rTreg) ratios were analyzed in relation to *IL17A* rs2275913 genotype in patients with MASLD (n = 58), including 31 with histology-proven MASH, and 28 healthy controls.

**Results:**

We identified the *IL17A* rs2275913 minor allele variant as a risk factor for fibrosis progression in patients with MASLD. In patients with advanced fibrosis, we also observed an increased abundance of fungal CTG species, including *Candida albicans* and *Debaryomyces hansenii*, which are potent triggers of Th17 responses. Integrating genetic predisposition with mycobiome composition, *ex vivo* T-cell stimulation assays demonstrated that donors carrying the *IL17A* rs2275913 minor allele secreted significantly higher levels of IL-17A in response to CTG species. Additionally, patients with MASH carrying the *IL17A* rs2275913 risk allele had elevated Th17/Treg ratios in peripheral blood.

**Conclusions:**

Genetic predisposition to enhanced Th17 responses, in the context of mycobiome dysbiosis, may promote MASH progression and liver fibrosis.

**Impact and implications:**

Liver inflammation and fibrosis are key drivers of the transition from bland steatosis to metabolic dysfunction-associated steatohepatitis (MASH). Our findings identify a combinatorial mechanism in which genetic predisposition to enhanced IL-17A signaling, together with gut mycobiome dysbiosis, promotes MASH development and fibrosis progression. This work highlights the importance of host–mycobiome interactions in shaping inflammatory liver disease and supports further investigation into targeted strategies aimed at modulating IL-17A–mediated immune responses in patients with MASLD. Such approaches may offer novel opportunities for risk stratification and therapeutic intervention.

## Highlights

• Genetic predisposition and gut mycobiome dysbiosis trigger BS-to-MASH transition.
• Patients with advanced liver fibrosis have elevated intestinal CTG species abundance.
• IL-17A secretion is dysregulated in patients with advanced liver fibrosis.
• The *IL17A* rs2275913 A/A variant is a genetic risk factor for fibrosis progression.

## Graphical abstract

**Figure:**
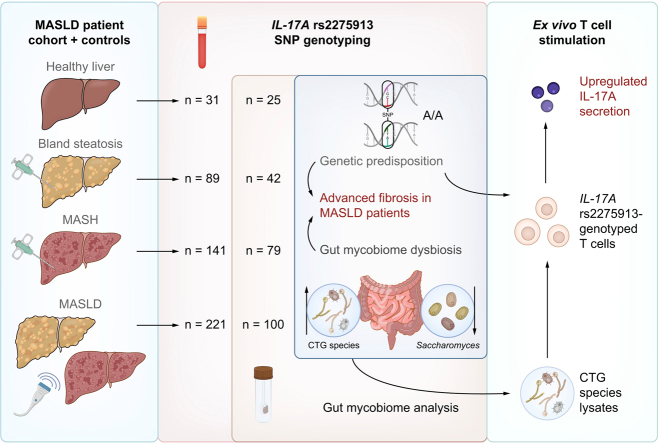

## Introduction

Metabolic dysfunction-associated steatotic liver disease (MASLD, formerly known as non-alcoholic fatty liver disease [NAFLD^1^]) is a leading cause of chronic liver diseases, with a global prevalence of approximately 25%.^2^ MASLD is characterized by excess fat accumulation in the liver without relevant alcohol consumption and is commonly associated with obesity, type 2 diabetes and metabolic syndrome.^3^ Fat accumulation in hepatocytes leads to bland steatosis (BS), the initial step in MASLD pathogenesis.^4^ Continued fat accumulation and lipotoxicity trigger inflammation and the transition to metabolic dysfunction-associated steatohepatitis (MASH) and ultimately to cirrhosis.^5^ The reasons why some patients progress to MASH and others do not remain unclear, but exaggerated T helper 17 cell (Th17) responses might be involved.^6^

The liver receives approximately 75% of its blood supply via the portal vein and therefore has a close connection to the human intestinal tract, which is densely colonized by microorganisms collectively referred to as the microbiome.^7^ Gut microbiota dysbiosis has been repeatedly observed in obesity and type 2 diabetes mellitus (DM).^8^^,^^9^ It was recently shown that the composition of gut microbiota also affects MASLD pathogenesis.[10], [11], [12], [13] In particular, short-chain fatty acid (SCFA)-producing bacteria such as *Fusobacteriaceae*, *Prevotellaceae*, and *Ruminococcaceae* might be involved.^14^

The role of intestinal fungi in MASLD is still poorly understood, likely due to several technical challenges.^15^^,^^16^ Especially the unresolved taxonomy of the polyphyletic genus *Candida* is problematic as it comprises relevant human gut mycobionts including the group of CTG species, which translate the CTG codon predominantly to serine instead of leucine. CTG species are of special importance in human gut colonization and comprise both opportunistic pathogens (*e.g. Candida albicans*) and non-pathogenic species (*e.g*. *Debaryomyces hansenii*).^17^ *C. albicans* is one of the most abundant human mycobionts and a major inducer of human antifungal Th17 cell responses.^18^

Recently, a distinct fecal mycobiome signature was identified in non-obese patients with MASLD, characterized by a high abundance of *Malassezia* spp. in patients with BS and increased abundance of *Candida albicans* and *Penicillium* spp. in patients with MASH. Notably, increased intestinal *C. albicans* colonization was also associated with increased levels of systemic antibodies against *C. albicans* as well as advanced fibrosis.^19^ Furthermore, the presence of *C. albicans*-specific T cells in the liver has been demonstrated in alcohol-associated liver disease (ALD).^20^

The aim of our study was to investigate the role of the gut mycobiota in MASH in the context of intrinsic variations in IL-17A signaling. Our results identify a novel *IL17A* genetic risk variant for liver fibrosis in MASH and indicate that intestinal colonization with *C. albicans* and related species (CTG species) may contribute to enhanced inflammation in the presence of this genotype.

## Patients and methods

Part of the methods are described in the supplementary file.

### Patients (MASLD cohort)

In this prospective study, 451 patients with MASLD were enrolled between 2016-2019 in the Division of Hepatology of the Department of Medicine II, University Hospital Würzburg, Germany. All study participants were >18 years old and diagnosed with MASLD by histology (n = 230) and/or clinically by transient elastography (TE; fibroscan and controlled attenuation parameter) (n = 350). We included all patients with clinically characterized MASLD in our cohort irrespective of histological characterization to investigate associations between genetic variations in antifungal immunity and gut mycobiome imbalance with the largest possible sample size. Although liver histology remains the gold standard for MASLD diagnosis, the more readily accessible technique of TE is widely used and validated, demonstrating high accuracy for both the diagnosis and exclusion of advanced fibrosis.^21^ Additionally, it reduces the risk of sampling error due to heterogeneous distribution of fibrosis when assessing liver biopsy specimens.^22^ As fibroscan is not considered the gold standard for diagnosing MASLD, we relied on histology-proven patient grouping whenever the sample size per group was sufficient, in order to minimize potential misclassification bias.

Clinical and anthropometric characteristics of the study cohort are shown in Table 1. A cut-off for daily alcohol consumption was set (<20 g/d for females and <30 g/d for males) and individuals with other underlying liver diseases (*e.g*., autoimmune liver disease or chronic viral hepatitis) were excluded. Information on patient’s last antibiotic treatment was documented. Medication like incretin mimetics led to exclusion from this study. As this represents a real-life patient cohort, dietary information could not be obtained for all participants. Fecal, serum and whole blood samples were immediately snap-frozen and stored in the local biobank.

**Table 1:** MASLD patient cohort characteristics.

	Patients with MASLD (n = 451)	Healthy controls (n = 31)
**General information**		
Male	166 (37.5%)	15 (48.4%)
Female	277 (62.5%)	16 (51.6%)
Age (years)	46.5 (18-73)	27.3 (23-37)
BMI (kg/m^2^)	46.2 (21.6-78.2), n = 450	21.4 (17.5-30)
Underweight (<18.5)	0	4 (12.9%)
Normal (18.5-24.9)	9 (2%)	23 (74.2%)
Overweight (25-29.9)	45 (10%)	3 (9.7%)
Obese – type I (30-34.9)	31 (7%)	1 (3.2%)
Obese – type II (35-39.9)	29 (6.5%)	0
Obese – type III (>40)	336 (74.5%)	0

**Liver function tests**		
AST (U/L)	36.8 (11-249), n = 450	20.5 (11.6-45.6), n = 27
ALT (U/L)	49.4 (5.8-469.7)	18.5 (10-46.6), n = 28
GGT (U/L)	65.2 (7.6-914), n = 450	NA
ALP (U/L)	77.2 (0-222), n = 450	NA
AST/ALT ratio	0.9 (0.2-3.7), n = 450	1.2 (0.6-1.6), n = 27
Glucose (mg/dl)	111.2 (70-444), n = 430	NA
Lipid metabolism		
Cholesterol (mg/dl)	187.5 (22-342), n = 419	NA
Triglyceride (mg/dl)	166.8 (31-1,188), n = 419	NA

**Elastography**		
Fibroscan (kPa)	11.6 (1.8-75), n = 350	NA
CAP (dB/m)	346.5 (40-400), n = 258	NA

**Comorbidities**		
Diabetes mellitus	195 (43.2%)	1 (3.2%)
Arterial hypertension	303 (67.2%)	0
Hyperlipidaemia	123 (27.3%)	0
Coronary heart disease	22 (4.9%)	0

Values are shown as n (%), or means (range).

ALP, alkaline phosphatase; ALT, alanine aminotransferase; AST, aspartate aminotransferase; CAP, controlled attenuation parameter; GGT, gamma-glutamyltransferase; MASLD, metabolic dysfunction-associated steatotic liver disease.

### Data visualization

Figures were generated by R software 3.6.3, using ggplot2 package.

### Statistics

Associations between the single nucleotide polymorphism (SNP) genotypes and fibroscan values, or grouped fibrosis status (cut-off 9.7 kPa), were investigated with generalized linear models adjusting for age, BMI, sex and *PNPLA3* rs738409 with the *glm* function of the R stats package. Due to its potential as a genetic risk factor for MASLD,^23^ we additionally adjusted for the *PNPLA3* rs738409 genotype in all SNP-based generalized linear model (glm) calculations. The *PNPLA3* rs738409 genotyping data were available for samples of our MASLD patient cohort and had been generated in a previous study.^24^ Statistical analysis of these data with the *glm* function confirmed primary findings from this study (Fig. 2C). To exclude potential bias induced by the chosen fibroscan of 9.7 kPa,^25^ we additionally repeated glm analysis using a cut-off of 10 kPa, which yielded similar results (p_glm_ = 0.043 (odds ratio = 1.64)).

Correlations between mycobiome and clinical data were assessed by Spearman’s correlation adjusting for age, sex, and obesity-related parameters (age, sex, BMI, DM, arterial hypertension [aHT] and hyperlipidemia) using the function *pcor.test* from R package ppcor. Differentially abundant genera were identified by the Wilcoxon rank-sum test using the R stats package, and by a glm adjusting for previously mentioned parameters (genus ∼ fibroscan.group + age + sex + BMI + DM + aHT + hyperlipidemia), with the *glm* function from the R stats package. The association between the presence or absence of CTG species and fibrosis state was calculated by the Fisher test, using the *fisher.test* function from the R stats package. A glm adjusting for previously mentioned parameters was used to study the association between CTG species and fibroscan value (genus ∼ fibroscan + age + sex + BMI + DM + aHT + hyperlipidemia), with the *glm* function from the R stats package. When exploring all data, the antibiotic intake was included for adjustment when appropriate.

## Results

### Study population

**Fig. 1:**
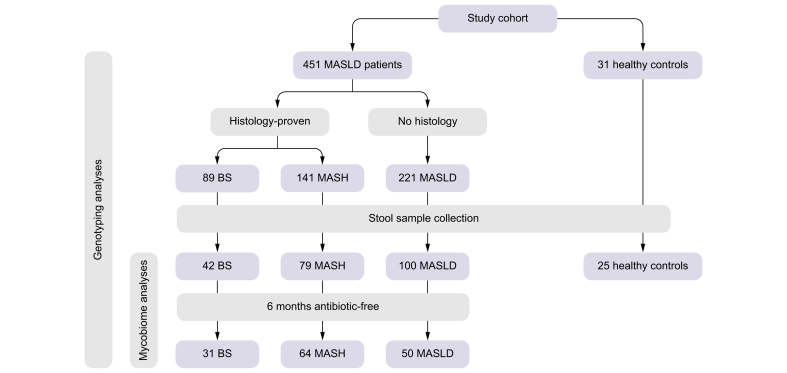
Flow diagram with an overview of the study participants. BS, bland steatosis; MASH, metabolic dysfunction-associated steatohepatitis; MASLD, metabolic dysfunction-associated steatotic liver disease.

A total of 482 European individuals were recruited for this study, including 230 with histology-proven MASLD (89 BS and 141 MASH). Fig. 1 illustrates the clinical and histological phenotypes of the study participants in a flow diagram. Stool samples were collected from a subcohort comprising 42 patients with BS, 79 with MASH, and 100 with MASLD without histological classification. Patients with and without a 6-month antibiotic-free interval were analyzed separately. In addition, a control group of healthy individuals (HC; n = 31) was included; all controls underwent rigorous clinical assessment to exclude any liver-related disease.

**Fig. 2:**
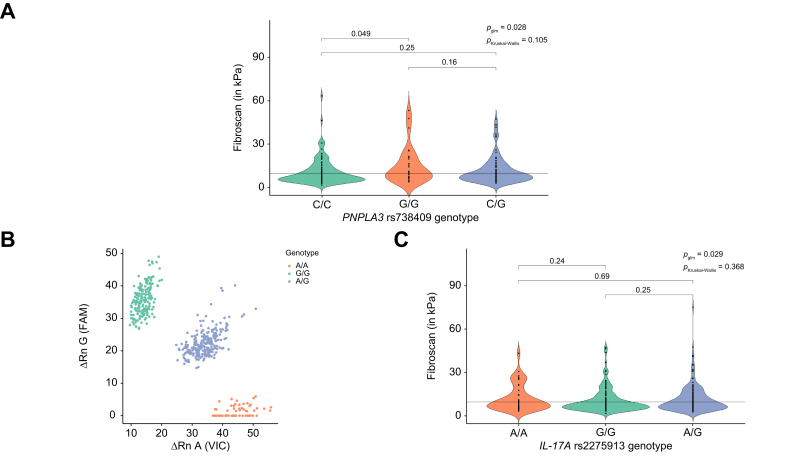
The *IL17A* rs2275913 genotype is associated with liver stiffness in MASLD. (A) Violin plot for visualization of known *PNPLA3* risk variant rs738409 association with fibrosis as assessed by fibroscan. Statistical comparison was performed using the Kruskal-Wallis test (*p*_Kruskal-Wallis_) and generalized linear models adjusted for age, sex and BMI (*p*_glm_) based on a fibroscan cut-off of 9.7 kPa. (B) Allelic discrimination plot after TaqMan SNP genotyping. (C) Violin plot for visualization of *IL17A* genotype association with fibrosis as assessed by fibroscan. Statistical comparison was performed using Kruskal-Wallis test (*p*_Kruskal-Wallis_) and generalized linear models adjusted for age, sex, BMI, *PNPLA3* rs738409 genotype (p_glm_) based on fibroscan cut-off = 9.7 kPa. MASLD, metabolic dysfunction-associated steatotic liver disease; SNP, single nucleotide polymorphism.

### Genetic variation in *IL17A* predisposes patients to develop fibrotic MASLD

Given the importance of IL-17A signaling in MASH-related inflammation, we sought to identify genetic factors in the form of SNPs governing Th17 activation in our patient cohort. We first validated the association between the PNPLA3 rs738409 genotype and liver fibrosis, as assessed by fibroscan values, in the available dataset, given that this variant is a major genetic risk factor for MASLD (Kruskal–Wallis: *p* = 0.105; glm adjusted *p* = 0.028; odds ratio = 2.58; Fig. 2A). Based on this validation, all subsequent SNP-based glm analyses performed in this study were adjusted for the *PNPLA3* rs738409 risk genotype.

Using available dbSNP databases, we identified three potential genetic variants in Th17 signaling-associated genes which could be linked to inflammatory gastrointestinal disease. However, a significant association with MASLD parameters was only found for the *IL17A* rs2275913 SNP and not for *CLEC7A* rs16910526 nor *CARD9* rs4077515 (Fig. S1). *IL17A* rs2275913 has previously been associated with inflammatory bowel disease.^26^ TaqMan SNP genotyping of our 451-patient MASLD cohort identified 175 G/G (homozygous for major allele variant, 38.8%), 55 A/A (homozygous for minor allele variant, 12.2%), and 221 A/G (heterozygous, 49%) genotypes (Fig. 2B). Genotype frequencies were in Hardy-Weinberg equilibrium and selection for specific genotypes was excluded (Fig. S2). The calculated minor allele frequency of 36.7% is comparable to the published ALFA European cohort minor allele frequency of 34.85%. Statistical analysis demonstrated a significant association between the IL17A rs2275913 genotype and liver fibrosis, as assessed by fibroscan (Kruskal–Wallis: *p* = 0.368; glm adjusted: *p* = 0.029; odds ratio = 1.7; Fig. 2C). Patients carrying the minor allele (A/A & A/G) showed increased liver stiffness and more severe fibrosis compared to those with a homozygous major allele genotype (G/G).

### A distinct mycobiome composition characterizes patients with MASH

Based on previous reports, mycobiome dysbiosis in the intestine can be linked to Th17 activation.^19^ Thus, we analyzed mycobiome composition using ITS1 libraries for 145 individuals from our study cohort to explore the possible association of fungi with MASLD progression and liver damage. On average, we generated 15,500 high-quality, non-chimeric reads per sample and fungal annotation identified 29 genera and 223 species in total. Genus-level fungal profiles showed that *Saccharomyces* (16.7%), *Penicillium* (16.1%), and CTG species (12.5%) were the most abundant fungal colonizers among our study participants. We used the CTG species group for genus clustering, including pathogenic (*C. albicans*, *C. tropicalis*, *C. dubliniensis*, *C. parapsilosis)* and non-pathogenic members (*D. hansenii).* Distantly related *Candida* species, such as *Nakaseomyces glabratus* (formerly *Candida glabrata),* were analyzed separately.^17^^,^^27^

In total, 76 patients in our cohort reported antibiotic use within the 6 months before stool collection. As a recent study showed that antibiotics may have a long-term influence on the mycobiome,^28^ we investigated whether antibiotics had a noticeable impact on the gut mycobiome profiles of the different disease groups. Indeed, the mycobiome alpha diversity measured by the Shannon and Simpson index at the genus level was significantly increased in individuals with MASH who had used antibiotics compared to those who had not (Wilcoxon rank-sum test: Shannon *p* = 0.028; Simpson *p* = 0.025; Fig. S3). However, no comparable effects were observed in the BS or MASLD groups when comparing antibiotic-exposed and antibiotic-free patients, nor were differences detected in beta diversity, as assessed by Aitchison distance, between antibiotic-exposed and antibiotic-free patients in any disease group (PERMANOVA adjusted for age, sex, and obesity-related parameters; *p* >0.05). Nevertheless, to minimize any potential impact of antibiotic use on downstream analyses, all primary analyses were performed using datasets restricted to long-term antibiotic-free samples, unless otherwise specified. Alternatively, mycobiome analyses were conducted using the full dataset with adjustment for antibiotic intake where appropriate (see Methods for details).

**Fig. 3:**
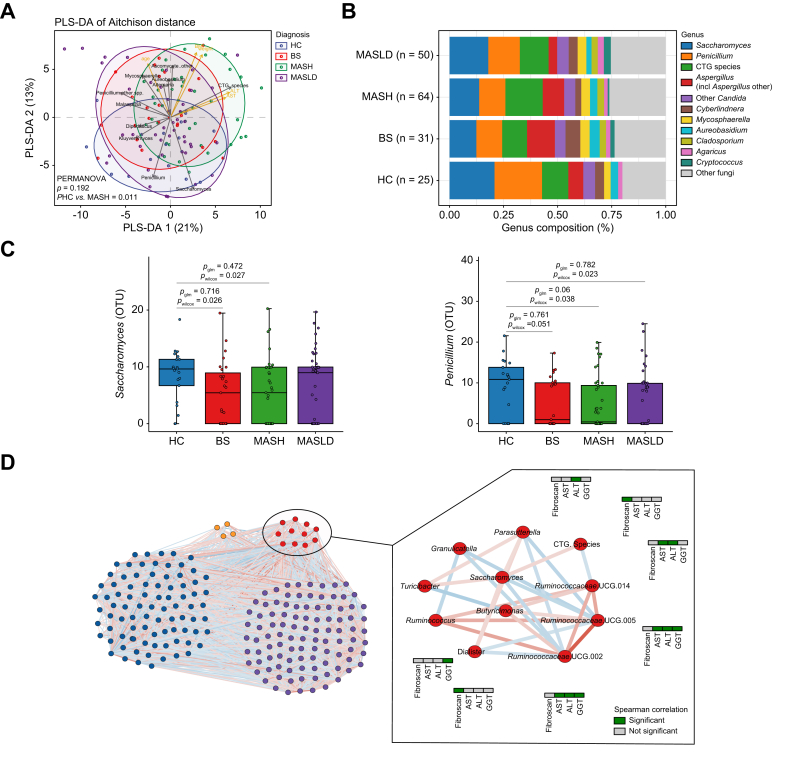
Mycobiome changes in the different diagnosed groups and healthy controls and microbial community network. (A) Beta diversity. PLS-DA of Aitchison distance of the mycobiome composition by diagnosis. (B) Overview of mycobiome composition at genus level in MASLD, BS, MASH, and HC groups. (C) Boxplot of *Saccharomyces* and *Penicillium* abundances. Statistical comparison between groups (HC, BS, MASH, and MASLD) was performed using Wilcoxon rank-sum test (*p*_wilcoxon_) and generalized linear models adjusting for age, sex, and obesity-related parameters (*p*_glm_). (D) Microbial community network showing the four subcommunity modules. Significant negative correlations are shown in blue and positive in red. The module significantly associated with MASLD-related parameters is shown with red nodes and significant correlations between the genera and fibroscan, AST, ALT, and GGT are shown in green. ALT, alanine aminotransferase; AST, aspartate aminotransferase; BS, bland steatosis; GGT, gamma-glutamyltransferase; HC, healthy controls; MASH, metabolic dysfunction-associated steatohepatitis; MASLD, metabolic dysfunction-associated steatotic liver disease; PLS-DA, partial least squares-discriminant analysis.

To study the mycobiome changes related to MASLD progression, we first performed pairwise comparisons between BS, MASH, MASLD and HC in alpha diversity measured by the Shannon and Simpson indexes and found no significant differences between the four groups (Wilcoxon rank-sum test, *p* >0.05 for all pair group comparisons for Shannon and Simpson index, data not shown). Beta diversity analysis using Aitchison distance to assess the overall mycobiome community differences showed that the fungal composition was significantly different between patients with MASH and HC (PERMANOVA adjusted for age, sex and obesity-related parameters, *p* = 0.01, Fig. 3A).

We then explored the differences in fungal abundance between the disease groups (BS, n = 31; MASH, n = 64; MASLD, n = 50) and HC (n = 25). In the HC group, the most abundant genus was Penicillium (22.2%), followed by Saccharomyces (20.9%) and CTG species (12.2%) (Fig. 3B). A similar abundance pattern was observed in the BS and MASLD groups, but not in MASH. In the MASH group, CTG species represented the most abundant fungal group (approximately 18%), followed by Saccharomyces (14.1%) and Penicillium (12.5%) (Fig. 3B). Overall, the relative abundance of *Saccharomyces* (Wilcoxon rank-sum test: HC *vs*. BS *p* = 0.026, HC *vs*. MASH *p* = 0.027) and *Penicillium* spp. (HC *vs.* BS *p* = 0.051, HC *vs*. MASH *p* = 0.038, HC *vs*. MASLD *p* = 0.023) was reduced in patients with BS and MASH compared with HC (Fig. 3C). However, these differences were no longer statistically significant after adjustment for age, sex, and obesity-related parameters in glm (adjusted *p* >0.05; Fig. 3C), indicating that these factors may confound the observed genus-level abundance differences.

We subsequently repeated all the analytical steps using the full cohort and obtained similar results as for the antibiotic-free set of samples although not all changes reached statistical significance (Fig. S4A).

Finally, we used 16S data from our cohort in order to build a microbial community network to identify possible associations between fungal and bacterial genera and MASLD progression. Using all cohort samples, we built a community network using FastSpar,^29^ and identified a total of 5,848 significant correlations (SparCC, *p* <0.05) from which 4,017 remained significant after multiple testing correction (false discovery rate correction, q <0.1). Using greedy modularity optimization, a total of four subcommunities were identified in the full network (Fig. 3D). We then studied the associations between these subcommunity modules and MASLD and identified one module consisting of two fungal (CTG species group and *Saccharomyces*) and nine bacterial (including *Ruminococcus*, *Dialister*, and *Parasutterella* amongst others) genera that was significantly associated with MASLD-related parameters (fibroscan, liver function tests) (Fisher’s exact test, *p* = 0.049, odds ratio = 3.580). These findings suggest an interplay between the fungal and bacterial kingdoms in the pathogenesis of MASLD.

### Increased abundance of CTG species in patients with advanced fibrosis

**Fig. 4:**
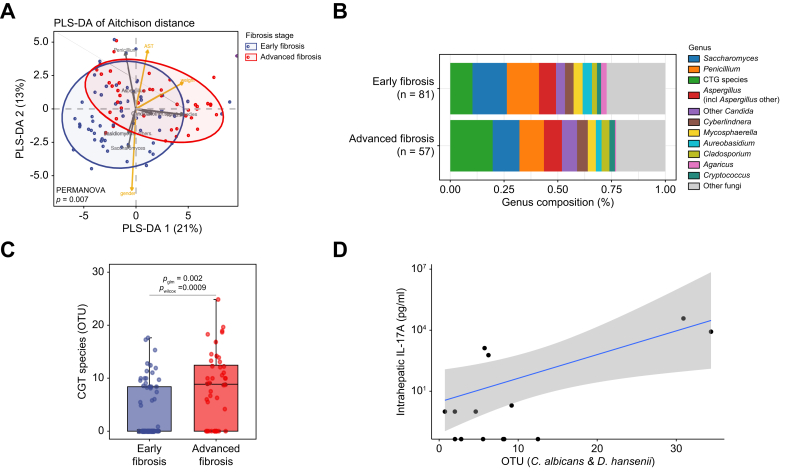
Mycobiome changes across fibroscan-based fibrosis groups and their correlation with intrahepatic IL-17A levels. (A) Beta diversity. PLS-DA of Aitchison distance of the mycobiome composition by fibrosis stage group. (B) Overview of mycobiome composition at genus level in early and advanced fibrosis groups (fibroscan cut-off </> 9.7 kPa). (C) Boxplot of CTG species abundances. Statistical comparison between early and advanced fibrosis was performed using Wilcoxon rank-sum test (*p*_wilcoxon_) and generalized linear models adjusting for age, sex and obesity-related parameters (*p*_glm_). (D) Intrahepatic IL-17A levels show a positive correlative trend with increased abundance of *C. albicans* and *D. hansenii* (Spearman coeeficient = 0.352, *p* = 0.218). OUT, operational taxonomic unit; PLS-DA, partial least squares-discriminant analysis.

To investigate whether changes in mycobiome composition are linked to progression of liver fibrosis, we classified the individuals into early or advanced fibrosis groups using a previously established fibroscan cut-off value of 9.7 kPa.^25^ The beta diversity analysis using Aitchison distance showed significant differences in the mycobiome composition between early and advanced fibrosis groups (PERMANOVA adjusted for age, sex, and obesity-related parameters, *p* = 0.007, Fig. 4A). Further analysis of the mycobiome composition (Fig. 4B) showed that CTG species were significantly more abundant in the advanced fibrosis group compared with the early fibrosis group, even after adjustment for age, sex, and obesity-related parameters (Wilcoxon rank-sum test: *p* = 0.0009; glm adjusted: *p* = 0.002; Fig. 4C).

To further corroborate our findings, we calculated beta diversity using Aitchison distance in the early and advanced fibrosis groups including all individuals, rather than restricting the analysis to antibiotic-free patients. Again, significant differences were identified (PERMANOVA adjusted, *p* = 0.01, Fig. S4B). A significant increase in CTG species abundance in advanced *vs.* early fibrosis (Wilcoxon rank-sum test: *p* = 0.0007; glm adjusted: *p* = 0.002) was also evident when analyzing the full cohort (Fig. S4C). Thus, in both antibiotic-free and total study cohorts, CTG species abundance is significantly higher in the advanced fibrosis group, suggesting that these species may contribute to disease progression.

Regression analysis between fibroscan liver stiffness values – independent of the arbitrary cut-off of 9.7 kPa – and CTG species abundance also revealed a significant relationship (glm adjusted for age, sex, and obesity-related parameters, *p* = 0.001, estimate = 0.22). Consistently, correlation analysis showed a significant positive correlation between fibroscan values and CTG species abundances (Spearman’s correlation adjusted, *p* = 0.026, ρ = 0.23). We also evaluated this association for the complete cohort and obtained similar results; the presence/absence of CTG species was associated with fibrosis stage (Fisher’s exact test: *p* = 0.002, odds ratio = 2.73), and CTG species abundances showed a positive correlation with fibroscan values (adjusted Spearman’s correlation: *p* = 0.01, ρ = 0.20 and glm adjusted, *p* = 0.002, estimate = 0.23, accounting for age, sex, obesity-related parameters and antibiotic use).

Finally, an increasing trend in CTG species abundance was also observed when samples were stratified by histology-defined fibrosis stage (Fig. S5). However, for advanced fibrosis stages (F3 and F4), the limited number of biopsied patients precluded statistical significance (Kruskal–Wallis test: *p* = 0.07 for the antibiotic-free dataset and *p* = 0.086 for the full cohort). To further investigate CTG species imbalance in advanced fibrosis, we analyzed the antibiotic-free sample set and identified a significant association between the presence or absence of the CTG species group and fibrosis stage (Fisher’s exact test: *p* = 0.006; odds ratio = 3.10).

To provide evidence for intrahepatic consequences of gut mycobiome dysbiosis, we correlated liver IL-17A levels available from our previous study^6^ with the abundance of the most prevalent CTG species, *C. albicans* and *D. hansenii*, obtained in the current study (Fig. 4D). Although intrahepatic IL-17A data for patients with MASLD included in the gut mycobiome analysis were available for only a small subset of the cohort, the results showed a trend toward a positive correlation between gut mycobiome dysbiosis and increased hepatic IL-17A levels (Spearman correlation = 0.352, *p* = 0.218).

### CTG species trigger increased proinflammatory responses in the presence of the *IL17A* rs2275913 risk genotype

**Fig. 5:**
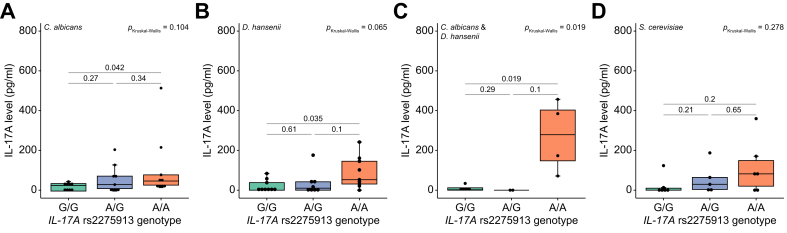
Increased IL-17A release from T cells from individuals homozygous for the rs2275913 minor allele variant. T cells were stimulated with fungal lysates and IL-17A concentrations in supernatants were measured by ELISA and calculated with a 4-parameter standard fit curve. 27 individuals were included in this assay. Due to interindividual variation of T cell numbers, not all stimuli were tested for each condition. IL-17A secretion after stimulation with (A) *C. albicans* lysate (G/G: n = 9, A/G: n = 9, A/A: n = 9), (B) *D. hansenii* lysate (G/G: n = 9, A/G: n = 8, A/A: n = 9), (C) *C. albicans* and *D. hansenii* lysate (G/G: n = 5, A/G: n = 2, A/A: n = 4) and (D) *S. cerevisiae* lysate (G/G: n = 6, A/G: n = 5, A/A: n = 6). Statistical comparisons for (A-D) were performed using Kruskal-Wallis test (*p*_Kruskal-Wallis_) and *t*-test comparing mean IL-17A values between genotypes. Horizontal lines in the boxplots indicate from top to bottom 75th percentile, median and 25th percentile. Whiskers display minimum and maximum values in 1.5x the interquartile range. Dots specify individuals for the three *IL17A* rs2275913 genotypes.

Next, we aimed to determine whether the *IL17A* rs2275913 SNP, associated with MASLD progression, could be linked to altered responses to fungal species that were identified in the dysbiotic mycobiome of patients with advanced liver fibrosis. We therefore stimulated freshly isolated T cells from rs2275913-genotyped donors *ex vivo* with fungal lysates. To ensure that differences in T-cell proportions among peripheral blood mononuclear cells of individual donors did not influence IL-17A levels, we first isolated T cells and then used equal numbers of T cells in the *ex vivo* stimulation assays. An age-dependent influence on CD4+ T-cell frequency was excluded, as the mean donor age was comparable across genotype groups (mean donor age 30-31 years). T cells were stimulated with fungal lysates from a pathogenic (*C. albicans*) and a non-pathogenic (*D. hansenii*) representative of the CTG species group,^30^^,^^31^ as well as with the non-CTG species *Saccharomyces cerevisiae*. Levels of multiple Th17 signaling-associated cytokines were quantified using Luminex technology. T-cell functionality was measured after stimulation with anti-CD3/anti-CD28 and did not show genotype-dependent differences, confirming that the observed effects induced by fungal lysates were species-specific. Both CTG species lysates triggered increased release of proinflammatory IFN-γ, TNF-α, IL-22 and IL-17A following *ex vivo* T-cell stimulation, especially in donors carrying the *IL17A* rs2275913 A allele (Table 2). Cytokine levels were generally lower after T-cell stimulation with the non-CTG species *S. cerevisiae* in comparison to *C. albicans* and *D. hansenii* (Table 2)*.* IL-17A release from stimulated T cells was additionally measured by highly sensitive ELISA. Again, effective and rs2275913 genotype-independent T-cell functionality was evaluated after stimulation with anti-CD3 (Fig. S6A), and all samples were normalized to the corresponding medium control values for each donor. T cells were stimulated with lysates of *C. albicans* and *D. hansenii* individually and in combination, as well as with the non-CTG species *S. cerevisiae* (Fig. 5). Both CTG species lysates induced IL-17A secretion, with T cells from individuals homozygous for the rs2275913 A allele (A/A) exhibiting significantly higher IL-17A levels compared with those from G/G and heterozygous donors (*C. albicans*: Kruskal–Wallis *p* = 0.104, A/A *vs.* G/G *p* = 0.042; *D. hansenii*: Kruskal–Wallis *p* = 0.065, A/A *vs.* G/G *p* = 0.035; Fig. 5A,B). Notably, this effect was further amplified when both fungal lysates were applied simultaneously at half concentration each (Kruskal–Wallis *p* = 0.019; A/A *vs.* G/G *p* = 0.019; Fig. 5C), indicating a cumulative antigenic effect, consistent with previous reports.^18^ The strongly elevated IL-17A secretion in donors with the rs2275913 A/A variant was not visible after T-cell stimulation with non-CTG species *S. cerevisiae* (Fig. 5D) or pathogenic *C. tropicalis*, *C. parapsilosis* and *N. glabratus* (Fig. S6B–D). As IL-17A and IL-17F are often coexpressed by Th17 cells,^32^ we additionally investigated the influence of the *IL17A* rs2275913 genotype on IL-17F expression (Fig. S7). Interestingly, IL-17F secretion trends mirrored those of IL-17A in relation to the underlying genotype but were significantly elevated only in donors with the rs2275913 A/A variant after co-stimulation with *C. albicans* and *D. hansenii* (Fig. S7C). Thus, the *IL17A* rs2275913 genotype modifies the amount of IL-17A produced in response to specific CTG species. Together with the increased abundance of CTG species in patients with MASLD, these findings suggest a combinatorial effect of genetically determined enhancement of Th17 responses and CTG species imbalance in driving fibrosis progression in patients with MASH.

**Table 2:** Cytokine levels after e*x vivo* stimulation of T cells from *IL17A* rs2275913-genotyped donors with *C. albicans*, *D. hansenii* and *S. cerevisiae*.

	C. albicans			D. hansenii			S. cerevisiae		
	G/G n = 9	A allele carrier n = 18	*p* value	G/G n = 9	A allele carrier n = 17	*p* value	G/G n = 6	A allele carrier n = 11	*p* value
IFN-γ	240.3 (0-1,661)	1,411 (0-4,825)	0.199	169.3 (0-717.2)	1,902.9 (0-7,833)	0.019	46.8 (0-211.2)	1,290.8 (0-6,155.3)	0.339
IL-17A	17.1 (0-49)	100.2 (0-680.9)	0.169	10.1 (0-45.7)	98.8 (0-635.6)	0.057	3.4 (0-20.2)	66.2 (0-315)	0.264
IL-22	52.1 (0-185.7)	253.5 (0-1275)	0.066	22.2 (0-95.8)	206.4 (0-1,038)	0.137	4.5 (0-21.3)	126.3 (0-648.2)	0.174
TNF-α	29.7 (0-79.2)	181 (0-861.1)	0.127	1.6 (0-7.2)	94.2 (0-405.7)	0.028	2.4 (0-11.4)	48.8 (0-282.9)	0.465

Values are shown as means and range.

### Patients with MASH carrying the *IL17A* rs2275913 A allele have elevated Th17/rTreg ratios

**Fig. 6:**
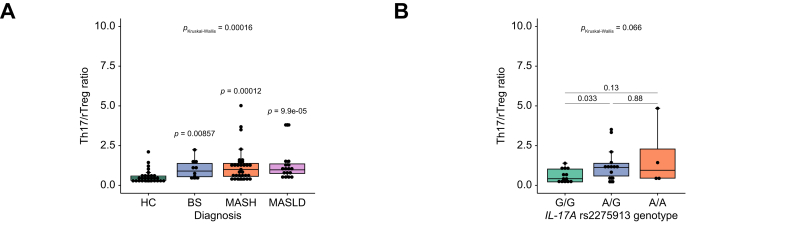
Elevated Th17/rTreg ratios in patients with MASH carrying the *IL17A r*s2275913 minor allele. (A) Th17/rTreg ratios in blood samples of HC and patients included in this study (HC: n = 28, BS: n = 10, MASH: n = 31 and MASLD: n = 17). (B) Th17/rTreg ratios in patients with MASH according to *IL17A* rs2275913 genotype (G/G: n = 13, A/G: n = 14, A/A: n = 4). Statistical comparisons were performed using Kruskal-Wallis test (*P*_Kruskal-Wallis_) and *t*-test comparing mean Th17/rTreg ratios using HC as a reference group (A) and between *IL17A* rs2275913 genotypes (B). Horizontal lines in the boxplots indicate from top to bottom 75th percentile, median and 25th percentile. Whiskers display minimum and maximum values in 1.5x the interquartile range. Dots specify individuals for the three *IL17A* rs2275913 genotypes. BS, bland steatosis; HC, healthy controls; MASH, metabolic dysfunction-associated steatohepatitis; MASLD, metabolic dysfunction-associated steatotic liver disease; rTreg, resting regulatory T cells; Th17, T helper 17 cell.

To further investigate whether carriers of the IL17A rs2275913 minor allele are predisposed to progress from MASLD to MASH, we analyzed the association between *IL17A* rs2275913 genotype and circulating Th17/resting regulatory T cell (Th17/rTreg) ratios. In previous work, we demonstrated elevated Th17/rTreg ratios in patients with MASH compared with HC, a finding that was also confirmed in the subset of patients included in the present study (MASH *vs.* HC: *p* = 0.00012; Kruskal–Wallis *p* = 0.00016; Fig. 6A). Genotyping of *IL17A* rs2275913 in these patients revealed significantly higher Th17/rTreg ratios in carriers of the A allele (A/G *vs*. G/G: *p* = 0.033; Kruskal–Wallis *p* = 0.066; Fig. 6B). Taken together with the results of the *ex vivo* stimulation assays, these findings suggest that patients with MASLD carrying the *IL17A* rs2275913 A allele may be predisposed to develop MASH through enhanced Th17 polarization and increased proinflammatory cytokine production. This effect is likely amplified in the context of elevated CTG species abundance in the gut, thereby contributing to liver inflammation and disease progression.

## Discussion

MASLD constitutes a major public health challenge worldwide. Its pathogenesis is influenced by multiple factors, including genetic predisposition, diet, and the composition of the intestinal microbiota.^33^ In this study, we identified a link between genetic variation in *IL17A* and gut mycobiome dysbiosis as contributing factors to inflammation-driven progression to MASH. Consistent with previous findings showing that the transition from BS to MASH correlates with increased IL-17A–producing intrahepatic CD4^+^ T cells and elevated Th17/rTreg ratios in peripheral blood,^6^ we found that patients carrying the *IL17A* rs2275913 minor allele (A/A genotype) had a higher risk of developing severe fibrosis, displayed elevated IL-17A secretion in response to fungal stimuli, and had higher Th17/rTreg ratios.

IL-17A plays an important role in maintaining health during immune responses to infection, injuries, and physiological stress, and is also crucial for the antifungal response of the adaptive immune system.^34^ However, dysregulation of IL-17A contributes to the development of various diseases, including liver fibrosis.^35^ Dysbiosis of the intestinal mycobiome also correlates with inflammatory diseases, including liver disease.^19^^,^^36^^,^^37^ Our observations indicate that both genetic variation in *IL17A* and increased intestinal abundance of CTG species may have a combined effect in fostering the progression of BS to MASH. This is in line with the previously described contribution of *C. albicans*-specific Th17 cells to ALD, indicating similar mechanisms for MASLD pathogenesis and specifically MASH development.^20^ In contrast, increased commensal gut colonization with *C. albicans* is positively correlated with systemic Th17-driven antifungal responses, which may enhance host defense against other pathogens.^38^ These findings underscore the importance of balanced antifungal IL-17A–mediated immunity for human health, which appears dysregulated in patients with MASLD carrying the *IL17A* rs2275913 A/A genotype, contributing to inflammation-driven liver fibrosis. Accordingly, future studies investigating targeted modulation of antifungal IL-17A responses may help identify novel therapeutic strategies. For example, ongoing studies, including our PINPOINT trial, are evaluating interventions with secukinumab^39^ to optimize IL-17A–mediated immune regulation and potentially mitigate liver inflammation in patients with MASLD.

The observed increase in intestinal CTG species in MASLD patients, particularly those with advanced fibrosis, mirrors the elevated abundance of *C. albicans* and *Debaryomyces* spp. reported in the gut of patients with alcohol use disorder.^37^ The *C. albicans*–secreted exotoxin candidalysin has been linked to disease severity in patients with ALD.^40^ Additionally, *C. albicans* strain diversity has been shown to influence immune responses in inflammatory bowel disease.^41^ Candidalysin might explain how high intestinal levels of *C. albicans* contribute to gastrointestinal and liver diseases. However, the gene encoding this peptide is absent in most non-*albicans* CTG species, including *D. hansenii*.^30^ Despite this, *D. hansenii* can trigger high levels of IL-17A secretion, similar to *C. albicans*. Different immune recognition mechanisms may exist among the various CTG species,^42^ but they ultimately converge on comparable Th17 activation patterns, which appear to drive the progression from BS to MASH and are likely amplified in carriers of the *IL17A* rs2275913 A/A genotype.

*D. hansenii* is often found on cheese and processed meat in the Western-style diet and is therefore often seen as a transient component of the mycobiome. Although the possible probiotic properties of *D. hansenii* have been extensively studied,^43^ its functional role in human disease remains poorly understood. In this study, *D. hansenii* exhibited a T cell–stimulatory capacity that was enhanced in patients carrying the *IL17A* rs2275913 A/A genotype. Previous studies have shown that *D. hansenii* contributes to impaired wound healing in patients with Crohńs disease and the corresponding mouse model.^44^ Notably, antifungal treatment with amphotericin B improved both wound healing and diet-induced liver fibrosis and steatohepatitis in mice with elevated intestinal abundance of *C. albicans* and/or *D. hansenii*.^19^^,^^44^ While the proinflammatory mechanisms of *D. hansenii* remain unclear, our co-stimulation data suggest a synergistic effect with *C. albicans* in driving Th17 responses in *IL17A* rs2275913 A/A donors, which warrants further investigation. Collectively, these findings indicate that non-pathogenic CTG species can contribute to gut- and liver-related inflammatory diseases, particularly in genetically predisposed *IL17A* rs2275913 A/A carriers.

Although our results corroborate the role of intestinal fungi, especially CTG species, and antifungal Th17 responses in MASLD pathogenesis, there are some limitations. Our ITS1-based gut mycobiome analysis clearly confirmed recent data generated by ITS2 sequencing. However, we cannot exclude that primer bias resulted in the omission of common mycobiome-associated species like *Malassezia* spp. in our dataset.^45^ Therefore, future studies should include an unbiased sequencing approach. Due to intestinal mycobiome variability between and even within individuals, future longitudinal studies are essential to exclude possible dietary, antibiotic or environmental effects. Such studies would further elucidate causal intestinal mycobiome changes associated with MASLD pathogenesis. Assessing diet-related changes in the gut mycobiome will be important in future studies, as, unlike prior studies of patients with MASLD and lower average BMI, we did not observe increased *Mucor* abundance, which has been associated with non-obese MASLD.^19^^,^^46^

Incorporating the bacterial influence on the interaction between CTG species and antifungal Th17 responses in MASLD pathogenesis would be of interest for future studies. Although the analysis of this complex triangle including human-fungal-bacterial interactions may be challenging under *in vitro* conditions, our interaction analysis already predicted that CTG species and SCFA-producing bacterial genera jointly contribute to liver pathology and MASLD progression. Bacterial-derived SCFAs might act as soluble mediators in this interactome as they have previously been linked to both MASLD pathogenesis and increased intestinal abundance of *C. albicans*.^47^^,^^48^ Interestingly, in our model the abundance of SCFA-producing bacteria was associated with increased aminotransferase values, indicating a metabolite-mediated effect on liver enzymes. Liver fibrosis, however, was only associated with increased CTG species abundance, supporting the hypothesis that these species induce dysregulated Th17 responses involved in liver fibrosis.

Overall, our results provide deeper insights into the role of intestinal fungi in MASLD pathogenesis, suggesting that a combination of genetically enhanced antifungal Th17 responses driven by the IL17A risk variant and elevated intestinal CTG species abundance promotes liver inflammation and fibrosis, thereby contributing to the progression from BS to MASH.

## Abbreviations

aHT, arterial hypertension; ALD, alcohol-associated liver disease; BS, bland steatosis; *C. albicans*, *Candida albicans*; DM, diabetes mellitus; *D. hansenii*, *Debaryomyces hansenii*; glm, generalized linear model; HC, healthy control; MASLD, metabolic dysfunction-associated steatotic liver disease; MASH, metabolic dysfunction-associated steatohepatitis; rTreg, resting regulatory T cells; *S. cerevisiae*, *Saccharomyces cerevisiae*; SCFA, short-chain fatty acid; SNP, single nucleotide polymorphism; TE, transient elastography; Th17, T helper 17 cell.

## Authors’ contributions

O.K., A.G., T.D. and G.P. conceived and designed the study. A.G., H.M.H., and M.R. recruited the participants and were responsible for clinical data collection. M.R. and H.M.H. collected fecal samples and extracted DNA from feces together with N.T.. A.M.A. and M.H. collected blood samples for the T cell stimulation assay, J.L. provided additional samples for this assay. H.M.H. and N.T. extracted DNA from blood and PBMC samples. R.M., N.E.N. and A.M.A. were involved in planning experimental analyses. N.T. performed and analyzed the experimental analyses. G.P., S.L.S., O.K. and M.H. were involved in planning of mycobiome analysis. S.L.S. and M.M. performed the metagenomics analyses. K.H. and A.Sh. performed the Luminex assays. A.Sch., N.R. and I.S.B. contributed to functional *ex vivo* assays. O.K., G.P., and A.G. led and supervised the research work. N.T. and S.L.S. wrote the manuscript. O.K., A.G., G.P., R.M., K.H. and M.R. edited the manuscript. All authors reviewed and made substantial contributions and approved the final version of the manuscript.

## Data availability

Raw sequences from ITS1 gene sequencing were registered at NCBI under BioProject PRJNA834619.

## Ethics approval & consent to participate

This study, involving the MASLD patient cohort (University of Würzburg: EK 96/12, 05.09.2012; EK 188/17, 13.01.2020) and healthy volunteers (University of Würzburg: EK 191/21, 16.08.2021) was approved by the local ethics committee and conforms to the ethical guidelines of the 1975 Declaration of Helsinki. We obtained written informed consent from all patients and healthy volunteers included in this study.

## Financial support

This project was funded by the IZKF Würzburg (project A-401), the 10.13039/100018694Marie Sklodowska-Curie Actions (MSCA), and Innovative Training Networks, H2020-MSCA-ITN-2018, 813781 “BestTreat” and by funds from the 10.13039/501100001659Deutsche Forschungsgemeinschaft (DFG) within the 10.13039/501100003383Collaborative Research Center CRC 124 FungiNet (project C3 to O. Kurzai; project B2 to T. Dandekar). This work was supported by the Deutsche Forschungsgemeinschaft (DFG, 10.13039/501100001659German Research Foundation) under the project "M3-NAFLD" (544831821). GP would like to thank the Deutsche Forschungsgemeinschaft (DFG, German Research Foundation) under Germany’s Excellence Strategy – EXC 2051 – Project ID 390713860.

## Conflict of interest

The authors report there are no competing interests to declare.

Please refer to the accompanying ICMJE disclosure forms for further details.
