# Supplementary material for: Mycobiome dysbiosis and genetic predisposition to elevated IL-17A contribute to fibrosis in MASLD
Source: JHEP Rep. 2025 Dec 23;8(3):101721. doi: 10.1016/j.jhepr.2025.101721 (PMC12914788; doi:10.1016/j.jhepr.2025.101721)
Supplement: Multimedia component 1 [file mmc1.pdf]

Mycobiome dysbiosis and genetic predisposition to elevated IL-17A contribute to fibrosis in MASLD

Nadja Thielemann, Sara Leal Siliceo, Monika Rau, Annika Schöninger, Nathalie Reus, Alexander M. Aldejohann, Aia Shehata, Isabell S. Behr, Natalie E. Nieuwenhuizen, Michaela Herz, Heike M. Hermanns, Mohammad Mirhakkak, Jürgen Löffler, Thomas Dandekar, Kerstin Hünniger-Ast, Ronny Martin, Gianni Panagiotou, Andreas Geier, Oliver Kurzai

Table of contents

Supplementary materials and methods……………………………………………………...2

Fig. S1……………………………………………………………………………………...6

Fig. S2……………………………………………………………………………………...7

Fig. S3……………………………………………………………………………………...8

Fig. S4……………………………………………………………………………………...9

Fig. S5……………………………………………………………………………………...10

Fig. S6……………………………………………………………………………….……11

Fig. S7……………………………………………………………………………………12

Supplementary references…………………………………………………………...…13

Supplementary materials and methods

DNA extraction from blood and PBMCs and TaqMan SNP Genotyping

DNA was extracted from frozen blood or PBMC samples using the Roche High Pure PCR Template Preparation Kit (Sigma Aldrich, #11796828001) and further used in TaqMan SNP Genotyping Assays (ThermoFisher, CN #4351376; CARD9 (ID: C__25956930_20), CLEC7A (ID: C__33748481_10), IL-17A rs2275913 (ID: C__15879983_10). Assays were conducted with the qTower3 (Analytik Jena) and analyzed with the qPCRsoft 3.4 software (Analytik Jena). The functionality of TaqMan SNP Genotyping was confirmed by additional sequencing of 5% samples and validating the obtained genotypes. For sequencing, a 414 bp part of interest in the IL17A gene was amplified (5´: ATATGATGGGAACTTGAGTAGTTTCCG, 3´: CTCCTTCTGTGGTCACTTACGTGG) with 2x Q5 polymerase master mix (NEB, #M0492L). PCR samples were purified with the PCR & Gel Clean-Up Kit (Macherey-Nagel, #740609.50) and sent to LGC Genomics for sequencing with the 5´ primer. DNA sequences were evaluated with ApE (v.3.0.8).

Fecal DNA extraction, internal transcribed spacer 1 and 16S rRNA sequencing

Microbial DNA was extracted from stool samples using the DNeasy PowerSoil Kit (Qiagen, #12888-100). Samples were divided into 4 subsamples to increase efficiency of the beat-beating step.

ITS sequencing using the Illumina platform Miseq V3 with paired-end reads of 300 bp was processed by LGC Genomics GmbH. The ITS1 region was amplified using ITS1F/ITS2R primers. The total read count was on average 54,000 reads/sample. 149 16S rRNA sequencing samples were processed by LGC Genomics GmbH using sequencing primers 341F-785R, targeting the V3-V4 region. The total read count was on average 56,000 reads/sample. Additionally, 97 16S rRNA sequencing samples from a previous study were processed as previously described [1].

Taxonomic profiling

Taxonomic annotation of fungal Internal Transcribed Spacer (ITS) was performed using the PIPITS pipeline [2] version 2.4, with default parameters including quality filtering, read-pair merging, ITS1 extraction and chimera removal. Remaining reads were binned based on 97% similarity as operational taxonomic units (OTUs) and aligned with QIIME [3] to the UNITE fungi database [4] using mothur classifier. Samples were then normalized by cumulative sum scaling using the R package metagenomeSeq. Fungi were generally grouped according to genus, except the *Candida* CTG species.

For the 16S rRNA sequencing data, quality control to remove low-quality reads and taxonomic annotation was performed using QIIME [3]. Raw reads were joined and trimmed with cutadapt to remove the primer sequences. Deblur workflow was used for filtering and denoising the joined reads. Assigning taxonomic information to each amplicon sequence variant (ASV) was performed using a Naive Bayes classifier with 99% similarity in QIIME. The classifier was fitted to the appropriate rRNA gene region (V3-V4) with the SILVA 132 database [5].

Diversity analysis

Alpha diversity indices detailing mycobiome community composition within samples were calculated using the R package vegan. Testing for significant differences in alpha diversity was performed using Wilcoxon rank-sum test. For estimating beta diversity reflecting community dissimilarities, cmultRepl function from R package zCompositions was first used to perform Bayesian-Multiplicative replacement of count zeros to the raw OTU table. Aitchison distances were calculated using aDist function from the R package robCompositions. We performed Partial Least Squares Discriminant Analysis (PLS-DA) using the mycobiome Aitchison distance matrix with the R package mixOmics. To test for significant differences in the mycobiome composition, permutational multivariate analysis of variance (PERMANOVA) as implemented in the function adonis from R package vegan adjusting for age, gender, obesity-related parameters (age, gender, BMI, DM, aHT and hyperlipidemia) was used. Mycobiome

community and clinical data (age, gender, height, weight, BMI, AST and ALT) were fit onto the ordination using the function envfit from vegan R package.

PBMC and T cell isolation

Freshly drawn blood from healthy volunteers was diluted 1:1 in PBS / 1 mM EDTA (Invitrogen, ThermoFisher Scientific, #AM9260G) containing 1% heat-inactivated human AB serum (Sigma Aldrich, #H4522-100ML) and separated via Biocoll density gradient medium (Bio&SELL, #BS.L 6115) in SepMate tubes (Stemcell Technologies, #85460) according to the manufacturer's instructions. Afterwards, PBMCs were washed 3 times with PBS-EDTA-human serum mix. As T cell proportions vary strongly between individual PMBC donors, we additionally isolated T cells before stimulation.

T cells were isolated from freshly isolated PBMCs by negative selection with the human Pan T Cell Isolation Kit (Miltenyi, #130-096-535) according to manufacturer's instructions and the purity of >90% was assessed by flow cytometry (Miltenyi MACSQuant®). To also include γδ T cells, which are known to be a major source of IL-17A, as well as IL17A-producing CD8 T cells [6], we used pan T cell isolation rather than isolating only $CD4^+$ T cells.

PBMC and T cell numbers were measured directly after isolation with the LUNA automated cell counter (Logos Biosystems) with a cell viability of >99% for each sample.

Preparation of fungal lysates

50 ml inoculated YPD medium (20 g/L glucose, 20 g/L peptone, 10 g/L yeast extract) was cultured overnight at 25 °C (*D. hansenii* CBS767) and 37 °C (*C. albicans* SC5314, *N. glabratus* CBS138, *C. parapsilosis* ATCC22019, *C. tropicalis* PI941, *S. cerevisiae* AR#0400). Overnight cultures were diluted 1:50 in 50 ml YPD medium and cultured for another 5 h. Cells were harvested by centrifugation at 4.000 x g for 10 min and the cell pellet was resuspended in lysis buffer (50mM Tris-HCl, 150 mM NaCl, 0.1 % Triton X-100, 1 mM DTT, 10 % glycerol) with freshly adjusted proteinase inhibitor (Sigma,

#S8820-20TAB). For lysis, 500 µl glass beads were added and samples underwent five cycles of 1min vortexing, each followed by a 1 min cooling step on ice. After centrifugation at 20.000 x g for 5 min, the supernatants were transferred to a new tube and stored in aliquots at -80 °C. The protein concentration was measured with the Qubit protein assay kit (Invitrogen, ThermoFisher Scientific, #Q33211).

Ex vivo T cell stimulation

Freshly isolated T cells were plated at 2 x 10^6 cells/well in 48-well plates and stimulated with 40 µg/ml fungal lysate or CTL-TestTM culture medium supplemented with PenStrep and L glutamine as medium control, in a final volume of 500 µl. T cell functionality was assessed by precoating wells with 1 µg/ml anti-human CD3 antibody (Miltenyi, #130-093-387) at 37 °C for 2 h before addition of cells and medium. All samples were supplemented with 1 µg/ml anti-human CD28 antibody (Miltenyi, #130-093-375). The plates were incubated for 48 h at 37 °C with 5 % CO_2. All samples were prepared in duplicates. After incubation, supernatants were frozen at -80 °C until cytokine measurement.

Quantification of cytokines by multiplex immunoassay

Cytokines (IL-17A, IFN-γ, IL-22, TNF-α) were measured in supernatants of *ex vivo* T cell stimulation assays using Luminex technology (ProcartaPlex™ Multiplex Immunoassay, Thermo Fisher Scientific) according to manufacturer´s instructions.

IL-17A and IL-17F ELISA

Antigen-specific IL-17A levels were measured in supernatants in duplicate using the IL-17A ELISA kit (Invitrogen, ThermoFisher Scientific, #BMS2017) according to the manufacturer's instructions. The standard curve was calculated from blank-curated mean standard values with a 4-parameter curve fit (R package dr4pl, v2.0.0) and used for calculation of IL-17A levels in blank-curated samples. To account

for medium-mediated activation effects, all samples were normalized to the corresponding medium control values for each donor. IL-17F cytokine measurement using the IL-17F ELISA kit (Invitrogen, ThermoFisher Scientific, #BMS2037-2) and data analysis were performed in exactly the same way that is described above for IL-17A.

Supplementary figures

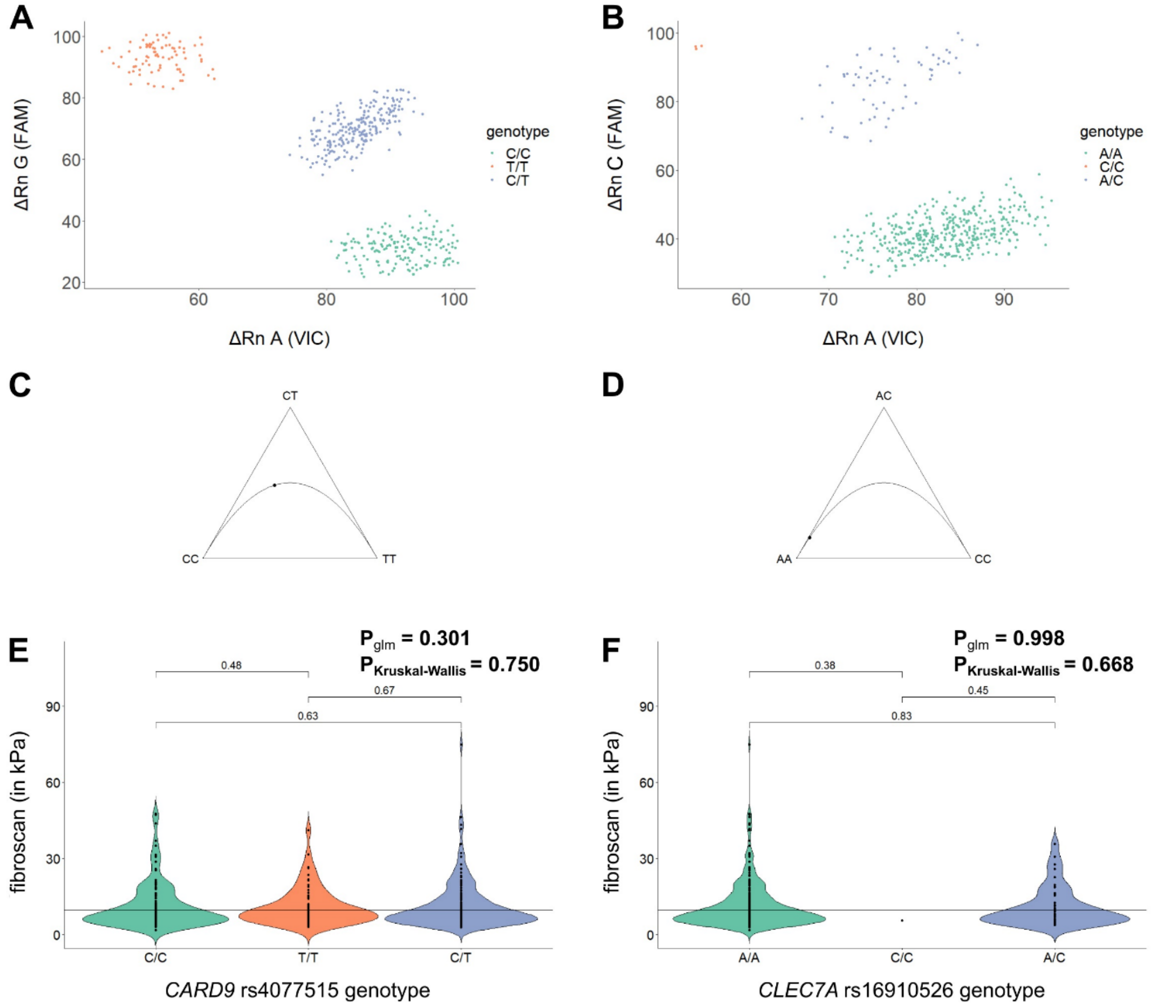

Fig. S1| TaqMan SNP genotyping data for *CARD9* rs4077515 and *CLEC7A* rs16910526. Allelic discrimination plots after genotyping for **A**) *CARD9* rs4077515 and **B**) *CLEC7A* rs16910526. Ternary Plot for evaluation of Hardy-Weinberg equilibrium for **C**) *CARD9* rs4077515 and **D**) *CLEC7A* rs16910526. Violin Plot for visualization of genotype association for **E**) *CARD9* rs4077515 and **F**) *CLEC7A* rs16910526 to fibroscan values. Statistical comparisons were performed using generalized linear models adjusted for age, gender, BMI, *PNPLA3* rs738409 genotype based on a fibroscan cut-off=9.7 kPa, but were not significant (p_{glm} (rs4077515)=0.3, p_{glm}(rs16910526)=0.5).

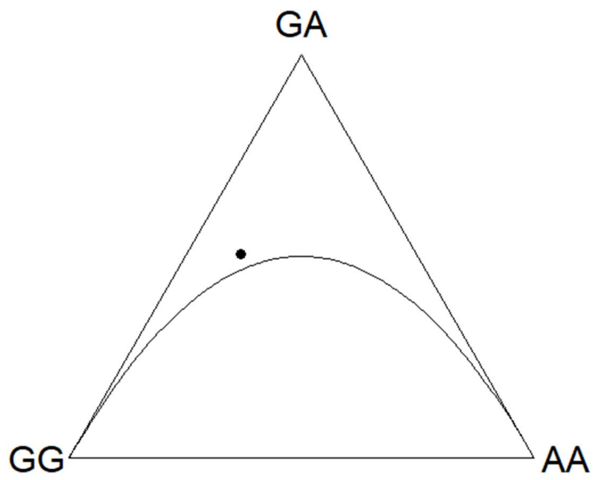

Fig. S2| Ternary Plot of *IL17A* rs2275913 data. *IL17A* rs2275913 genotyping data are in Hardy-Weinberg equilibrium and thereby selection for specific genotypes was excluded.

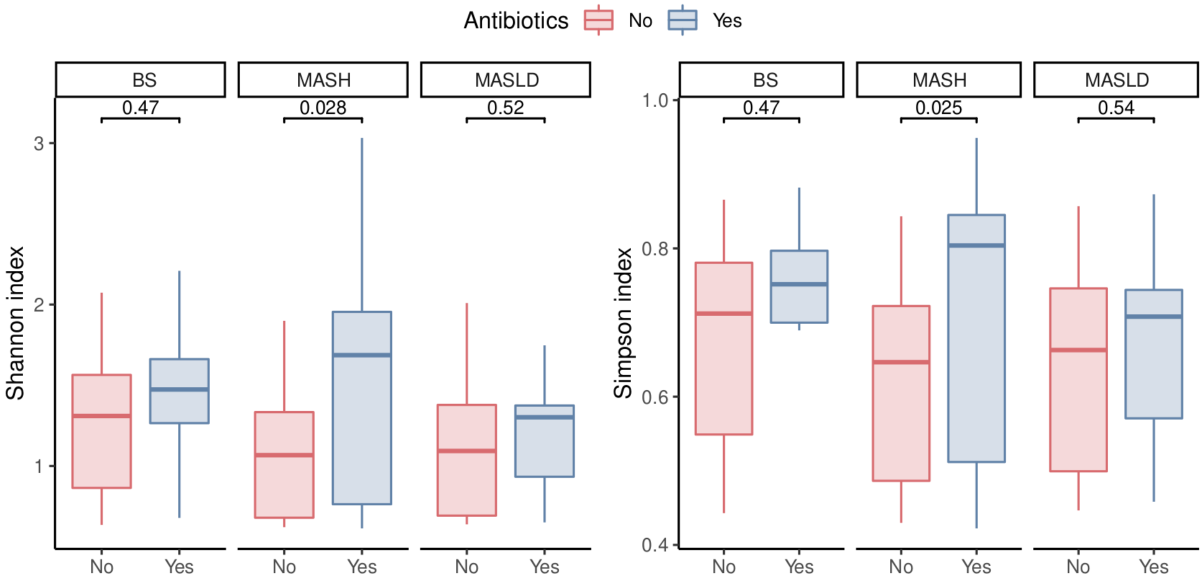

Fig. S3| Comparison of Shannon (left) and Simpson (right) indexes between antibiotic-free subjects (No, red) and subjects that used antibiotics within the six months prior to the sample collection (Yes, blue) in BS, MASH and MASLD groups.

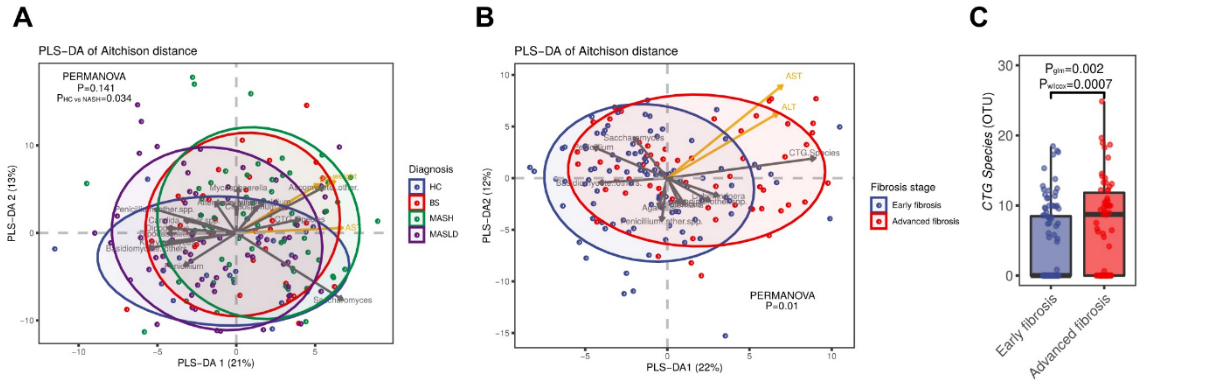

Fig. S4| Mycobiome changes using the full cohort of samples. **A**) Beta diversity. PLS-DA of Aitchison distance of the mycobiome composition by diagnosis. **B**) Beta diversity. PLS-DA of Aitchison distance of the mycobiome composition by fibrosis stage group. **C**) Boxplot of CTG species abundances. Statistical comparison between early and advanced fibrosis was performed using: Wilcoxon rank-sum test ($p_{wilcoxon}$) and generalized linear models adjusting for age, gender and obesity-related parameters and antibiotic intake (p_{glm}).

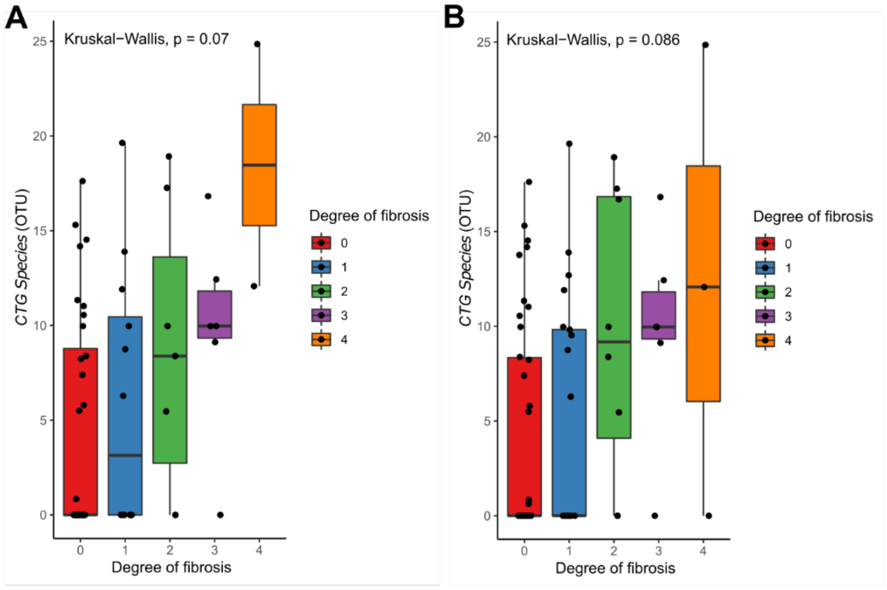

Fig. S5| Boxplot of CTG species abundances. **A**) Antibiotic-free set of samples. **B**) Full cohort. Statistical comparison between fibrosis stages (obtained by biopsy) in **A** and **B** were performed using Kruskal-Wallis test.

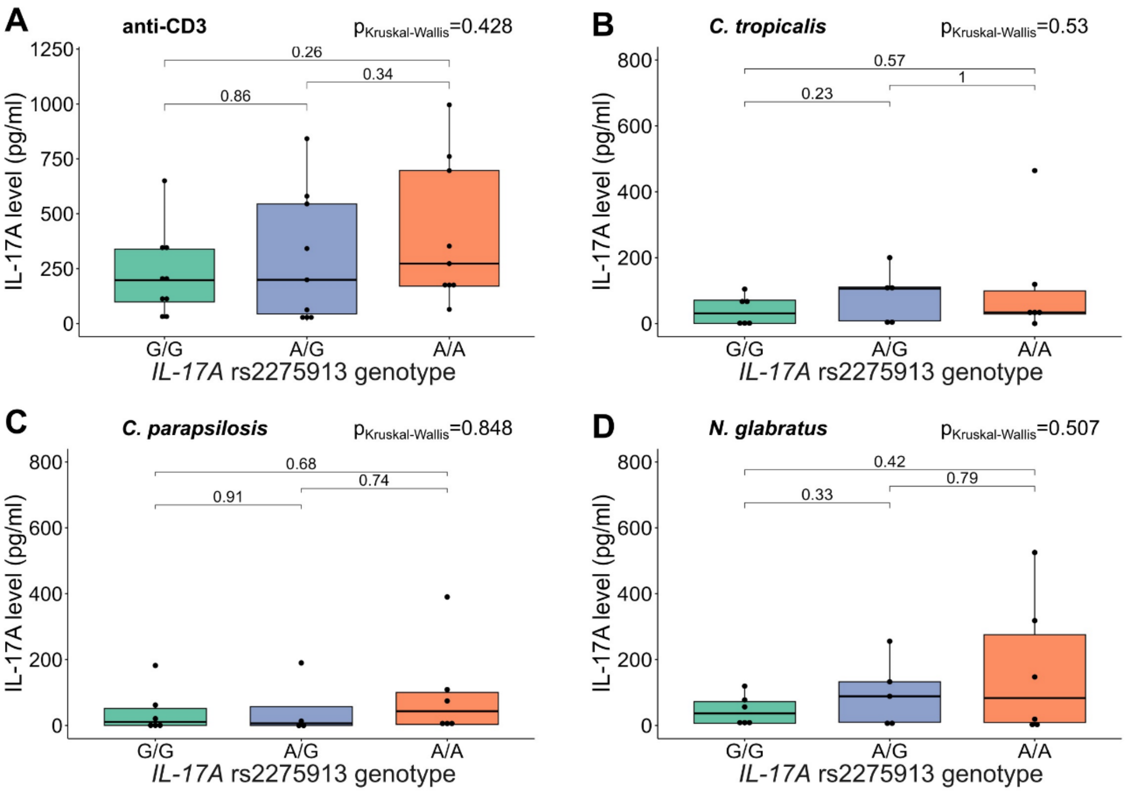

Fig. S6| IL-17A secretion of T cells after stimulation with anti-CD3 as a control and fungal lysates. IL-17A concentrations in supernatants were measured by ELISA and calculated with a 4-parameter standard fit curve. 27 subjects were included in this assay. Due to interindividual variation of T cell numbers not all stimuli were tested for each condition. **A**) IL-17A secretion after stimulation with anti-CD3 as control for sufficient T cell activation in all analyzed samples (G/G: *n=9*, A/G: *n=9*, A/A: *n=9*). **B-D**) IL-17A secretion after stimulation with **B**) *C. tropicalis* lysate (G/G: *n=6*, A/G: *n=5*, A/A: *n=6*), **C**) *C. parapsilosis* lysate (G/G: *n=6*, A/G: *n=4*, A/A: *n=6*) and **D**) *N. glabratus* lysate (G/G: *n=6*, A/G: *n=5*, A/A: *n=6*). Statistical comparisons for **A-D** were performed using Kruskal-Wallis Test ($p_{Kruskal\text{-}Wallis}$) and *t*-test comparing mean IL-17A values between genotypes. Horizontal lines in the boxplots indicate from top to bottom 75th percentile, median and 25th percentile. Whiskers display minimum and maximum values in 1.5x the interquartile range. Dots specify individuals for the three *IL17A* rs2275913 genotypes.

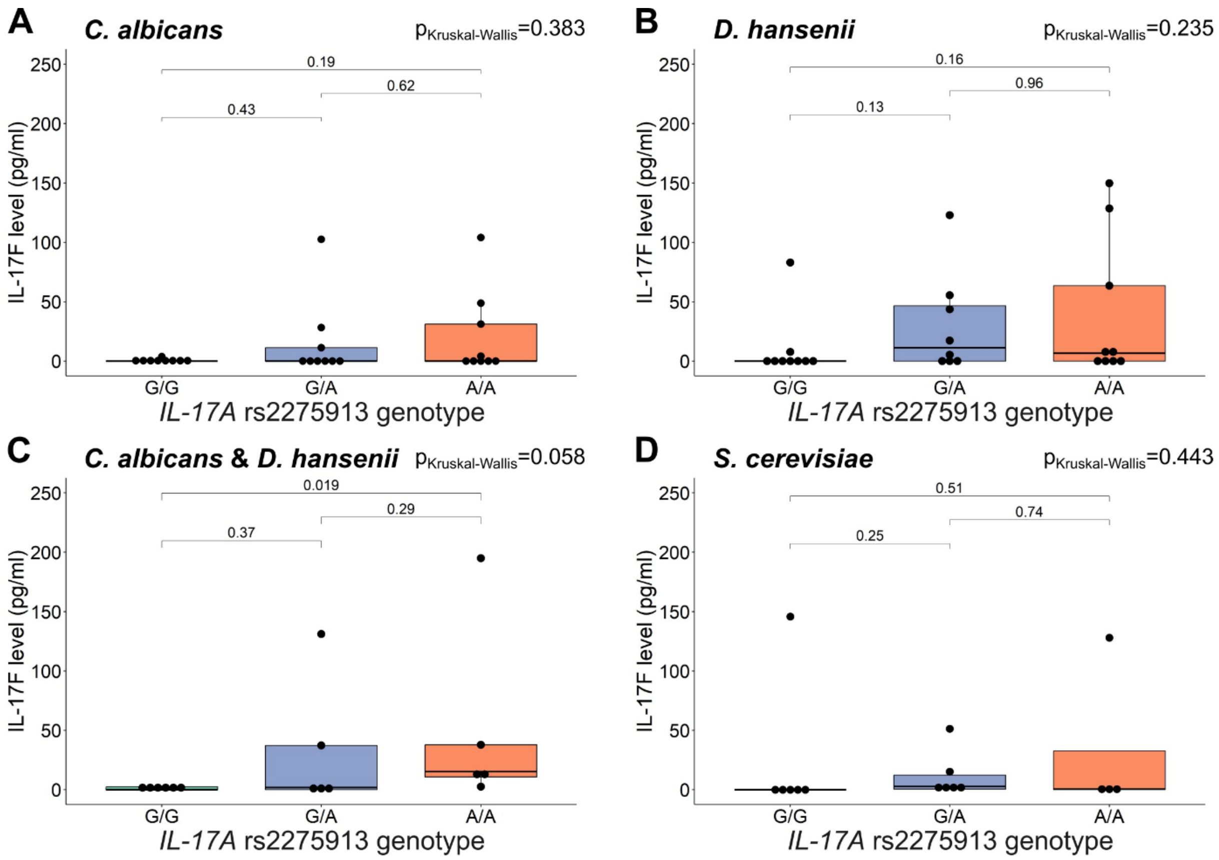

Fig. S7| IL-17F secretion of T cells after stimulation with fungal lysates. IL-17F concentrations in supernatants were measured by ELISA and calculated with a 4-parameter standard fit curve. 27 subjects were included in this assay. Due to interindividual variation of T cell numbers not all stimuli were tested for each condition. IL-17F secretion after stimulation with **A**) *C. albicans* lysate (G/G: *n=9*, A/G: *n=9*, A/A: *n=9*), **B**) *D. hansenii* lysate (G/G: *n=9*, A/G: *n=8*, A/A: *n=9*), **C**) *C. albicans* and *D. hansenii* lysate (G/G: *n=6*, A/G: *n=5*, A/A: *n=5*) and **D**) *S. cerevisiae* lysate (G/G: *n=6*, A/G: *n=6*, A/A: *n=4*). Statistical comparisons for **A-D** were performed using Kruskal-Wallis Test ($p_{Kruskal\text{-}Wallis}$) and *t*-test comparing mean IL-17F values between genotypes. Horizontal lines in the boxplots indicate from top to bottom 75th percentile, median and 25th percentile. Whiskers display minimum and maximum values in 1.5x the interquartile range. Dots specify individuals for the three *IL17A* rs2275913 genotypes.

Supplementary references

1 Rau, M. *et al.* Fecal SCFAs and SCFA-producing bacteria in gut microbiome of human NAFLD as a putative link to systemic T-cell activation and advanced disease. *United European Gastroenterol J* **6**, 1496-1507 (2018). https://doi.org:10.1177/2050640618804444

2 Gweon, H. S. *et al.* PIPITS: an automated pipeline for analyses of fungal internal transcribed spacer sequences from the Illumina sequencing platform. *Methods Ecol Evol* **6**, 973-980 (2015). https://doi.org:10.1111/2041-210X.12399

3 Caporaso, J. G. *et al.* QIIME allows analysis of high-throughput community sequencing data. *Nat Methods* **7**, 335-336 (2010). https://doi.org:10.1038/nmeth.f.303

4 Nilsson, R. H. *et al.* The UNITE database for molecular identification of fungi: handling dark taxa and parallel taxonomic classifications. *Nucleic Acids Res* **47**, D259-D264 (2019). https://doi.org:10.1093/nar/gky1022

5 Quast, C. *et al.* The SILVA ribosomal RNA gene database project: improved data processing and web-based tools. *Nucleic Acids Res* **41**, D590-596 (2013). https://doi.org:10.1093/nar/gks1219

6 van de Veerdonk FL, Netea MG. T-cell Subsets and Antifungal Host Defenses. *Curr Fungal Infect Rep* **4**: 238-243 (2010). https://doi: 10.1007/s12281-010-0034-6.
